# Supplementary material for: Biochemical analyses reveal amino acid residues critical for cell cycle-dependent phosphorylation of human Cdc14A phosphatase by cyclin-dependent kinase 1
Source: Sci Rep. 2018 Aug 8;8:11871. doi: 10.1038/s41598-018-30253-8 (PMC6082843; doi:10.1038/s41598-018-30253-8)

Biochemical analyses reveal amino acid residues critical for cell cycle-dependent phosphorylation of human Cdc14A phosphatase by cyclin-dependent kinase 1

Sara Ovejero[1,3], Patricia Ayala[1], Marcos Malumbres[4], Felipe X. Pimentel-Muiños[1], Avelino Bueno[1,2], and María P. Sacristán[1,2,]*

[1]Instituto de Biología Molecular y Celular del Cáncer (IBMCC), Universidad de Salamanca-CSIC, Campus Miguel de Unamuno, 37007 Salamanca, Spain.

[2]Departamento de Microbiología y Genética, Universidad de Salamanca, Campus Miguel de Unamuno, 37007 Salamanca, Spain.

[3]Current affiliation: Institute of Human Genetics, CNRS UMR 9002, CNRS, Université de Montpellier, 34396 Montpellier, France.

[4]Centro Nacional de Investigaciones Oncológicas (CNIO), E-28029 Madrid, Spain.

*Corresponding author:

María P. Sacristán: msacristan@usal.es

Supplementary Information

Supplementary Figure S1. Characterization of U-2-OS cell lines stably expressing HA-Cdc14A or the phosphatase dead form HA-Cdc14A(PD). (a) HEK293T cells were transfected with Flag-tagged wild-type Cdc14A or its inactive form Cdc14A(PD). After 24 hours of transfection cellular extracts were obtained and ectopic protein expression was detected by immunoblotting with anti-Flag antibodies. **(b)** Comparison of Cdc14A expression levels among U-2-OS cells stably expressing retrovirally transduced HA-Cdc14A or HA-Cdc14A(PD), and transient transfection with expression vectors containing HA-Cdc14A under the control of the CMV promoter (C: U-2-OS parental cells). Full-length Cdc14A blot is shown in Supplementary Fig. S15. **(c)** FACS analysis of control U-2-OS cells and the U-2-OS-HA-Cdc14A cell lines. To check the mitotic index, cells were processed to detect phospho-histone H3 (Serine 10). **(d)** Subcellular localization of Cdc14A or Cdc14A(PD) in stable cell lines. Cells were fixed and co-stained with anti-HA tag and anti-γ-tubulin antibodies. DNA was stained with DAPI. **(e)** U-2-OS-HA-Cdc14A cells were synchronized at G1/S by a double thymidine treatment and then released into fresh medium to allow progression through G2 phase. After 10 hours from release, rounded mitotic cells were selected by shake off. Cellular extracts were obtained from both asynchronous and mitotic cells, resolved by Phos-tag gels and analyzed by immunoblot using anti-Cdc14A antibodies. Full-length Cdc14A blot is shown in Supplementary Fig. S15.

Supplementary Figure S2. Cdc14A interacts with PP2A. Cellular extracts were obtained from U-2-OS cell lines stably expressing HA-Cdc14A (Figure S1) and Cdc14A was immunoprecipitated with anti-HA antibodies. Interaction with the endogenous PP2A α-catalytic subunit was detected by immunoblotting with specific anti-PP2A antibodies. WCE: whole cell extracts. Unprocessed original scans of blots are shown in Supplementary Fig. S15.

Supplementary Figure S3. Alignment of Cdc14 family members. Amino acid sequence alignment of Human Cdc14A, Mouse Cdc14A, *Xenopus* Cdc14a, *Saccharomyces cerevisiae* Cdc14 and *Schizosaccharomyces pombe* Cdc14/SpFlp1. Sequence alignment was performed using Constrain-based Multiple Alignment Tool.
(COBAL program (https://www.ncbi.nlm.nih.gov/tools/cobalt/re_cobalt.cgi). Shadowed in gray are indicated Cdk1 phosphorylation sites described in *S. pombe* and *S. cerevisiae*. All consensus motifs for Cdk1 phosphorylation on human Cdc14A (T110, T254, S411, S453, S511, S549 and T590) are indicated. Yellow marks indicate the Cdk1-targeted phosphorylation sites of human Cdc14A.

Supplementary Figure S4. Cdc14A phosphorylation by Cdk1 does not inhibit its phosphatase activity. **(a)** Cdc14A was immunoprecipitated from asynchronous or mitotic nocodazol arrested U-2-OS cells stably expressing Flag-Cdc14A, and its phosphatase activity was determined on pNPP substrate. Cdc14A(PD), immunoprecitated from asynchronous U-2-OS cells stably expressing Flag-Cdc14A(PD), was used as a negative control. Reactions were performed in triplicate. Data are representative of three independent experiments. **(b)** Wild-type Cdc14A, and Cdc14A-3A and -3E mutants were obtained by immunoprecipitation from U-2-OS cells stably expressing the corresponding Flag-Cdc14A version, and their phosphatase activity was determined on pNPP substrate. Reactions were performed in triplicate. Data are representative of three independent experiments. **(c)** HEK293T cells were co-transfected with Flag-KIBRA and EGFP-tagged wild-type Cdc14A, Cdc14A-3A, Cdc14A-5A or Cdc14A-5E mutants. After 12 hours of transfection, half of the cells were treated with nocodazole during 12 hours. Then, cellular extracts were obtained from both asynchronously growing or nocodazole treated cells, and analyzed by immunoblot with the indicated antibodies. Full-length blots are shown in Supplementary Fig. S15.

Supplementary Figure S5. Cdc14A phosphorylation by Cdk1 does not affect its association with interphase centrosomes. U-2-OS cells were transfected with EGFP-tagged Cdc14A or the phosphorylation mutants Cdc14A-3A or Cdc14A-3E. After 24 hours of transfection, cells were fixed and immunostained with antibodies against γ-Tubulin. DNA was stained with DAPI. Images were acquired using a Zeiss Axioimager Apotome microscope (objective 40X) and processed using Zeiss Zen lite software. (Scale bar: 10μM).

Supplementary Figure S6. Release of Cdc14A from centrosomes does not depend on Cdk1 phosphorylation. U-2-OS cells were transfected with EGFP-tagged Cdc14A or the phosphorylation mutants Cdc14A-3A or Cdc14A-3E. After 48 hours of transfection, cells were fixed and immunostained with antibodies against γ-Tubulin. DNA was stained with DAPI. Representative mitotic cells are shown. Images were acquired using a Leica DM6000B microscope (objective 40X) and processed using OpenLab 4.0.3 software. (Scale bar: 10μM).

Supplementary Figure S7. Phosphorylation of Cdc14A does not significantly affect its cellular stability. Derivatives of HeLa cell line, conditionally expressing low levels of Flag-tagged Cdc14A (WT), Cdc14A-3A (3A) or Cdc14A-3E (3E) in a tetracycline-inducible manner, were incubated with medium containing Doxycycline during 24 hours to induce ectopic Cdc14A expression. Cells were then treated with the inhibitor of protein synthesis cycloheximide and harvested at the indicated time points to check Cdc14A protein levels by immunoblot with anti-Cdc14A antibodies. Full-length Cdc14A blots are shown in Supplementary Fig. S16.

Supplementary Figure S8. Co-immunoprecipitation analysis of Cdc14A and Cdc14A-3A mutant. (a) HEK293T cells were co-transfected with HA-Cdh1 and Flag-tagged wild-type Cdc14A, its inactive form Cdc14A(PD) or Cdc14A(PD)-3A mutant. After 12 hours of transfection, cells were treated with nocodazole for 12 hours. Cellular extracts were immunoprecipitated with anti-Flag antibodies and analyzed by immunoblot to detect the presence of the two proteins. Asterisks show cross-reacting bands. **(b)** HeLa cell lines conditionally expressing Flag-tagged wild-type Cdc14A or Cdc14A-3A mutant, were incubated with medium containing Doxycycline to induce protein expression and with or without nocodazole during 12 hours. Cellular extracts were obtained and Cdc14A forms were immunoprecipitated with anti-Flag antibodies. Interaction with endogenous Plk1 was analyzed by immunoblotting. Unprocessed original scans of blots are shown in Supplementary Fig. S16.

Supplementary Figure S9. Full-length immunoblots relating to Figure 1 are shown.
Supplementary Figure S10. Full-length gels and immunoblots relating to Figure 2 are shown.
Supplementary Figure S11. Full-length gels and immunoblots relating to Figure 3 are shown.
Supplementary Figure S12. Full-length gels and immunoblots relating to Figure 4 are shown.
Supplementary Figure S13. Full-length gels and immunoblots relating to Figure 5 are shown.
Supplementary Figure S14. Full-length immunoblots relating to Figure 6 are shown.
Supplementary Figure S15. Full-length immunoblots relating to Supplementary Figures S1, S2 and S4 are shown.
Supplementary Figure S16. Full-length immunoblots relating to Supplementary Figures S7 and S8 are shown.

Supplemental Figure S1

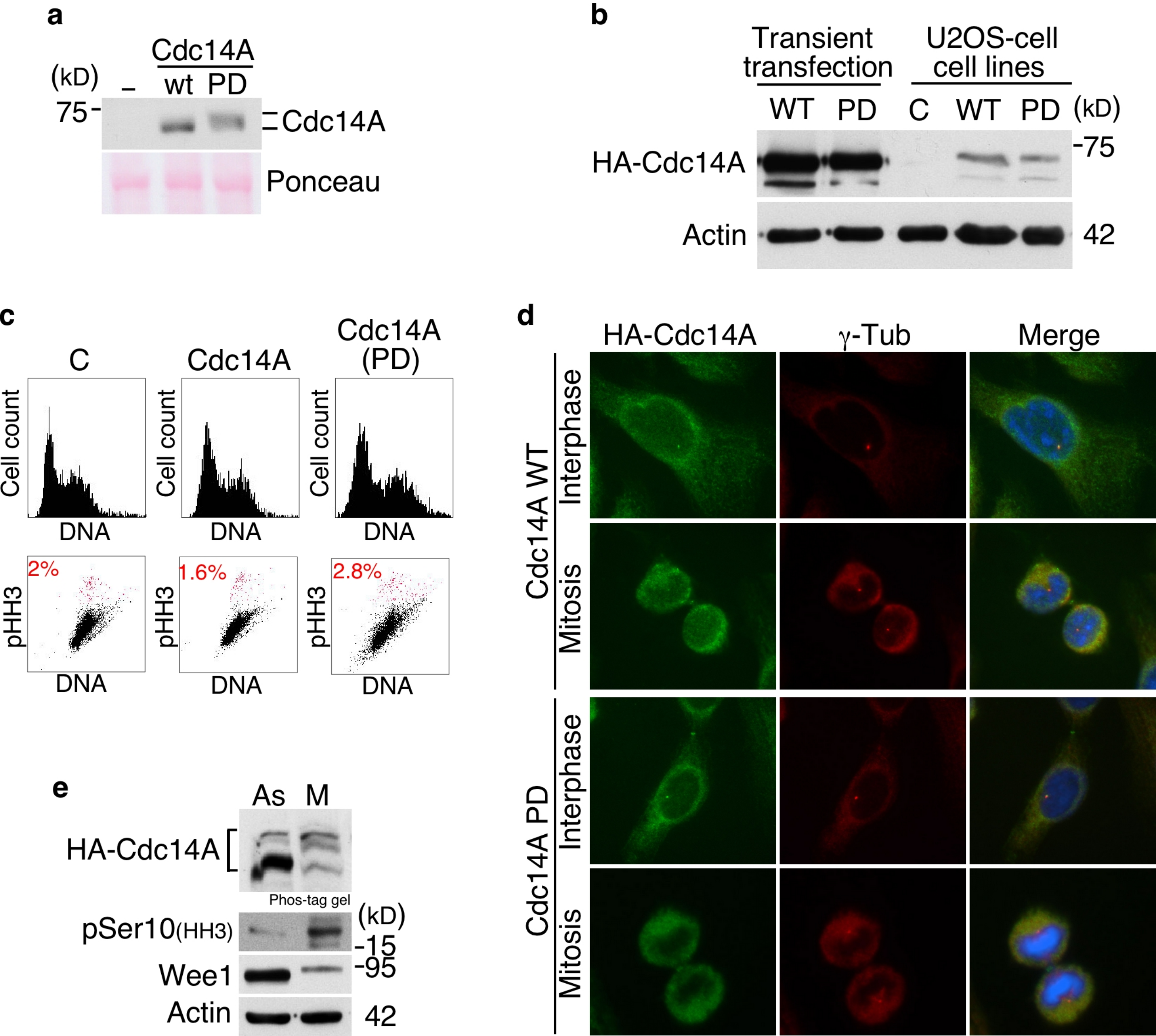

Supplementary Figure S2

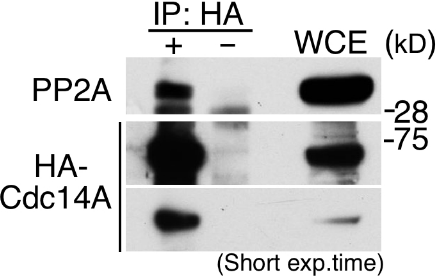

Supplemental Figure S3

```
                                                            T(110)
HsCDC14A  1    MAAES---GELIGACEFMKDRLYFATLRNRPK-ST-VNTHYFSIDEELVYENFYADFGPLNLAMVYRYCCKLNKKLKSYS  75
MmCDC14A  1    MAAES---GELIGACEFMKDRLYFATLRNRPK-ST-INIHYFSIDEELVYENFYADFGPLNLAMVYRYCCKLNKKLKSYS  75
XlCDC14A  1    M--DN---QELISASEVIKDRLYFAILRNKPK-ST-LNTHYFCTDEEFVYENFYADFGPLNLAQLYRYCCKLNKKLKSFS  73
SpFlp1    1    MDYQDdglGEMI---EFLEDKLYYTSLSQPPKaELyPHMHFFTIDDELIYNPFYHDFGPLNVSHLIRFAVIVHGIMGKHR  77
ScCDC14   1    MRRSVy-lDNTI---EFLRGRVYLGAYDYTPE-DT-DELVFFTVEDAIFYNSFHLDFGPMNIGHLYRFAVIFHEILNDPE  74

HsCDC14A  76   LSR--KKIVHYTCFDQRKRANAAFLIGAYAVIYLKKTPEEAYRALLSGSNPPYLPFRDASFGNCTYNLTILDCLQGIRKG  153
MmCDC14A  76   LSR--KKIVHYTSFDQRKRANAAFLIGAYAVIYLKKTPEEAYRALLSGSNPPYLPFRDASFGNCTYNLTVLDCLQGIRKG  153
XlCDC14A  74   LSR--KKIVHYTSFEQRKRSNAGFLISAYAVIYLKKSPEEVYRALLSGSNAQYLPFRDASFGSCTYNLTILDCLQGIRKA  151
SpFlp1    78   QAKksKAIVLYSSTDTRLRANAACLLACYMVLVQNWPPHLALAPLAQ-AEPPFLGFRDAGYAVSDYYITIQDCVYGLWRA  156
ScCDC14   75   NAN--KAVVFYSSASTRQRANAACMLCCYMILVQAWTPHQVLQPLAQ-VDPPFMPFRDAGYSNADFEITIQDVVYGVWRA  151

HsCDC14A  154  LQHGFFDFETFDVDEYEHYERVENGDFNWIVPGKFLAFSGP---HPKSKI-ENGYPLHAPEAY---FPYFKKHNVTAVVR  226
MmCDC14A  154  LQHGFFDFETFDAEEYEHYERVENGDFNWIVPGKFLAFSGP---HPKSKI-ENGYPLHAPEAY---FPYFKKNNVTTIVR  226
XlCDC14A  152  LHFGFFNFENFDADEYEHYERVENGDFNWIVPGKFLAFSGP---HPKSKI-ENGYPLHAPEAY---FPYFRKHNIRAVIR  224
SpFlp1    157  RESSILNIRNIDVHDYETYERVENGDFNWISP-KFIAFASP---IQAGWNhASTRPKKLPQPFaivLDYFVANKVKLIVR  232
ScCDC14   152  KEKGLIDLHSFNLESYEKYEHVEFGDFNVLTP-DFIAFASPqedHPKGYL--ATKSSHLNQPFksvLNFFANNNVQLVVR  228
                                                T(254)
HsCDC14A  227  LNKKIYEAKRFTDAGFEHYDLFFIDGSTPSDNIVRRFLNICEN-T--EGAIAVHCKAGLGRTGTLIACYVMKHYRFTHAE  303
MmCDC14A  227  LNKKIYEAKRFTDAGFEHYDLFFIDGSTPSDNIVRRFLNICEN-T--EGAIAVHCKAGLGRTGTLIACYVMKHYRFTHAE  303
XlCDC14A  225  LNKKIYDAKRFTDAGFDHYDLFFVDGSTPSDGIVRRFLNLCEN-T--DGAIAVHCKAGLGRTGTLIACYIMKHYRFTHSE  301
SpFlp1    233  LNGPLYDKKTFENVGIRHKEMYFEDGTVPELSLVKEFIDLTEE-VeeDGVIAVHCKAGLGRTGCLIGAYLIYKHCFTANE  311
ScCDC14   229  LNSHLYNKKHFEDIGIQHLDLIFEDGTCPDLSIVKNFVGAAETiIkrGGKIAVHCKAGLGRTGCLIGAHLIYTYGFTANE  308

HsCDC14A  304  IIAWIRICRPGSIIGPQQHFLEEKQASLWVQGDIFRSKLKNRPSSEGSINKILSGLDDMSIGGNLSKTQNMERFGEDNLE  383
MmCDC14A  304  IIAWIRICRPGSIIGPQQHFLKEKQASLWVQGDIFRSKLKNRPSSEGSITKIISTLDDMSIGANLSKLQSTERIGENNFE  383
XlCDC14A  302  TIAWIRTCRPGSIIGPQQHFLEEKQTWLWLQGDLHRSNQKQFQSEDRAVSRILTDFEELSVSGRFSSNNIYERDREQNNS  381
SpFlp1    312  VIAYMRIMRPGMVVGPQQHWLHINQVHF--RAYFY-EKAMGRAIQQATAAEPLATPP-----------------------  365
ScCDC14   309  CIGFLRFIRPGMVVGPQQHWLYLHQNDF--REWKYTTRISLKPSEAIGGLYPLISLEEY-------------RLQKKKLK  373
                                   S(411)                                     S(453)
HsCDC14A  384  DDDVEMKNGITQGDKLRALKSQRQPRTSPSCAFRSD-DTKGHPRAVSQPFRL--SSSLQGSAVTLKTSKMALSPSATAKR  460
MmCDC14A  384  DEDMEIKNNVTQGDKLRALKSQRHPRSSPSCAFRSD-DMKGHQRAMAQTFRL--SSSPQPTMSTMKTSKVCLSPSVTAKK  460
XlCDC14A  382  EDELDHSNGLTQGDKLRALKSRRHPRSATTGALRLD-DVKLHTRSTSQPFRVfaSNSTQGSMSPLKSSRVTSSTSAVGRR  460
SpFlp1    366  ----------------------RHPLNATNGTSQSNiSTPLPEPTPGQPRKV--SGHNPPSARRLPSASSVKFNEKLKNA  421
ScCDC14   374  DDKRVAQNNI-EGE-LRDLT--MTPPSNGHGALSAR--------NTSQPSTA------NNGSNSFKSSAVPQTSPGQPRK  435
                                                          S(511)
HsCDC14A  461  INRTSLSSGATVRSFSINSRLASSLGNLNAATDDPENKKTSSSSKAGFTASPFTNLLNGSSQPTTRNYPELNNNQYNRSS  540
MmCDC14A  461  ISRGSLSSGANIRSFSINSRLASSLGNLNAGTEEPETKKTTSLTKAAFIASPFTSFLNGSTQTPGRNYPELNNNQYTRSS  540
XlCDC14A  461  VAQSPRSTNSKGGGFSANLRFAGSLGNLNSALEEIESTRRPYTNRAISSQAVPSNLYHSHSRV--RSQGELNNNR-----  533
SpFlp1    422  SKQSIQNENKASYSSYEDSEIQNDDETRTVGTPTETISVVRLRRSSSQSNIEPNGVRSPTSSPTGSPIRRTSGNRWSSGS  501
ScCDC14   436  GQNGS------------------NTIEDINNNRNPTSHANRKVVIESNNSDDESMQDTNGTSNH----YPKVSRKKNDISS  494
                     S(549)                                  ..T(590)..
HsCDC14A  541  NSN----[2]NLNSPPGPhSAKTEEHTTILRPSYTG-lSSSSARFLSRSIP[29]KFNSAKEAF  623
MmCDC14A  541  NSNSSSS[6]NLNSSPVPqSAKPEEHTTILRPSFPGslSSSSVRFLSRSIP    SLQSEYVHY  603
XlCDC14A  534  -------   NQNVPSTP-----KDMYGSFRPSYTG--LASSPRFLSRSIP    SLQSEYAQI  576
SpFlp1    502  SHSKKSA   -QRSVSMS-SLNNTSNGRVAKPKPSK-----------SRLIS    ---------  537
ScCDC14   495  ASSSRME[1]--NEPSAT-NINNAADDTILRQLLPK-----------NRRVT[17]SIKK-----  551
```

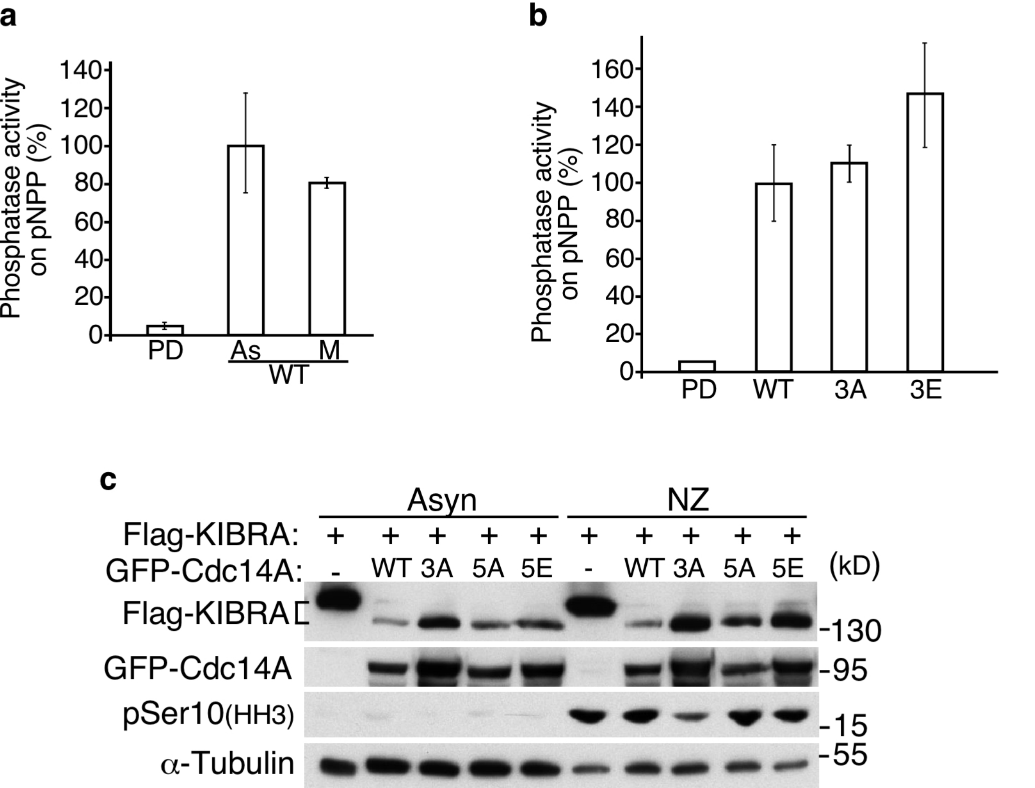
a
Phosphatase activity on pNPP (%)
140
120
100
80
60
40
20
0
PD
As
M
WT
b
Phosphatase activity on pNPP (%)
160
140
120
100
80
60
40
20
0
PD
WT
3A
3E
c
Asyn
NZ
Flag-KIBRA:
GFP-Cdc14A:
-
WT
3A
5A
5E
(kD)
Flag-KIBRA
GFP-Cdc14A
pSer10(HH3)
α-Tubulin
-130
-95
-15
-55

Supplemental Figure S5

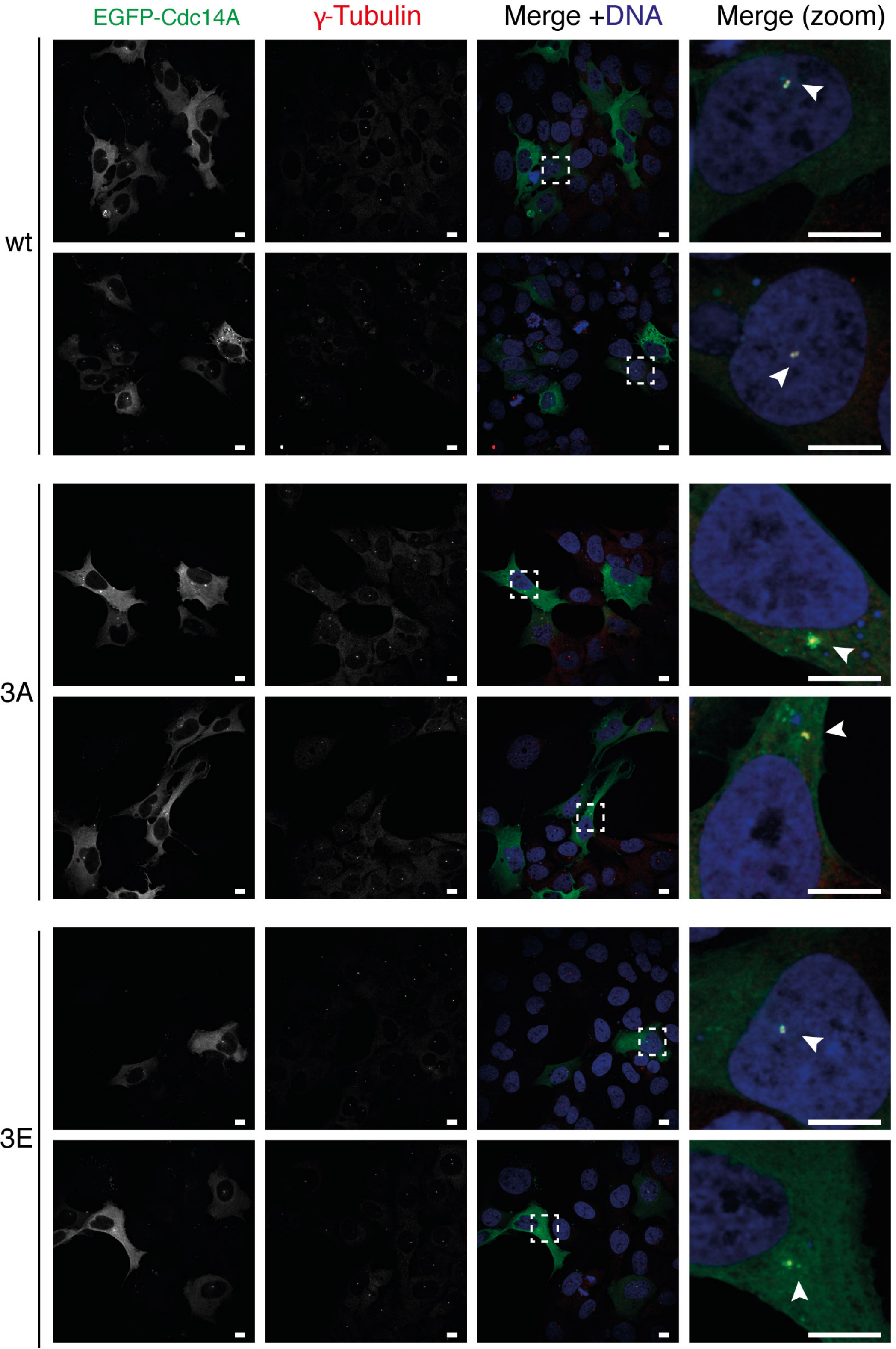

Supplemental Figure S6

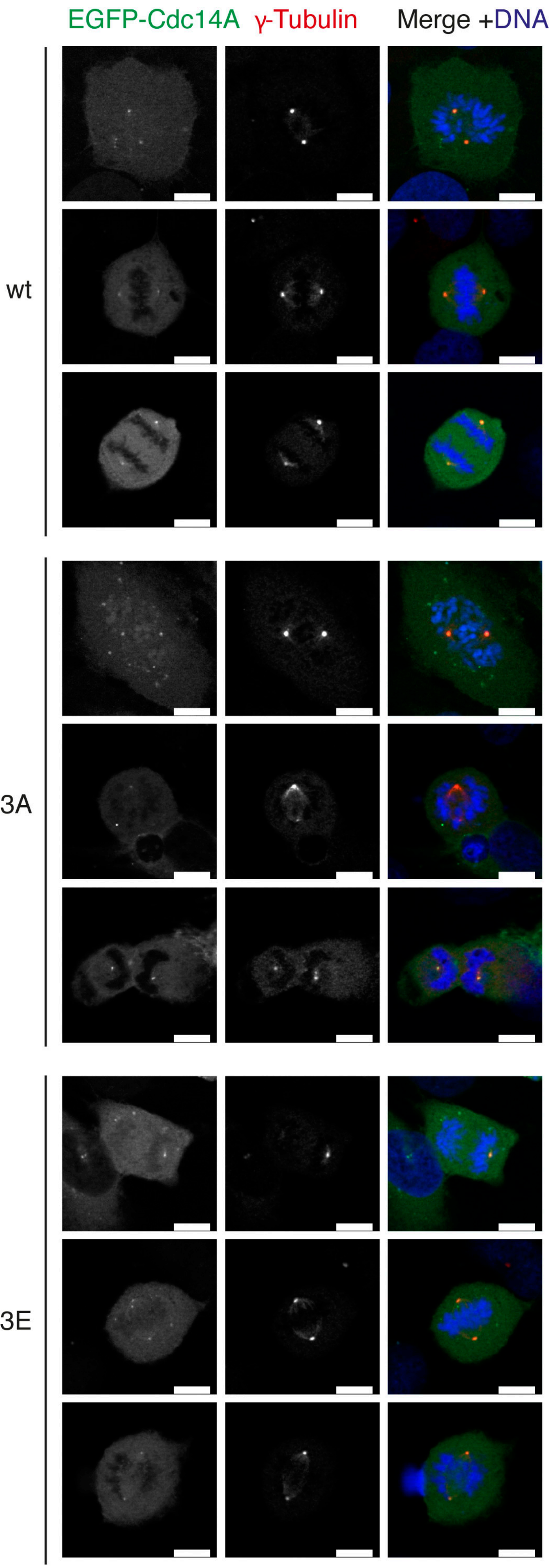

Supplemental Figure S7

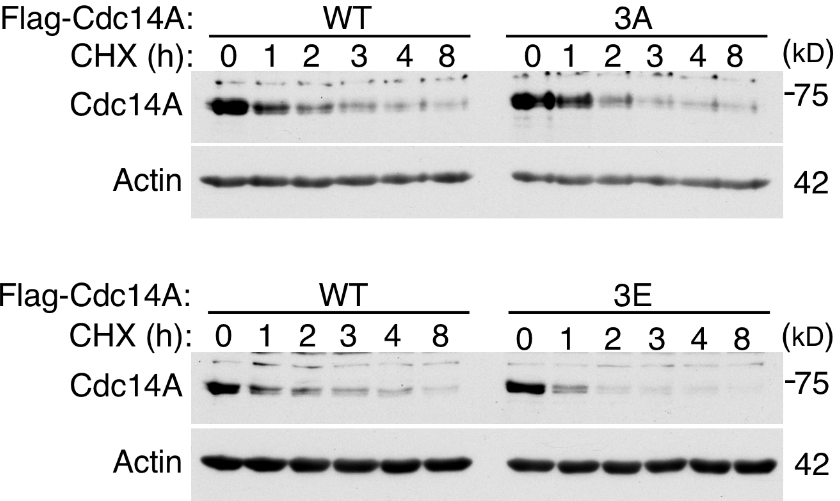

Supplemental Figure S8

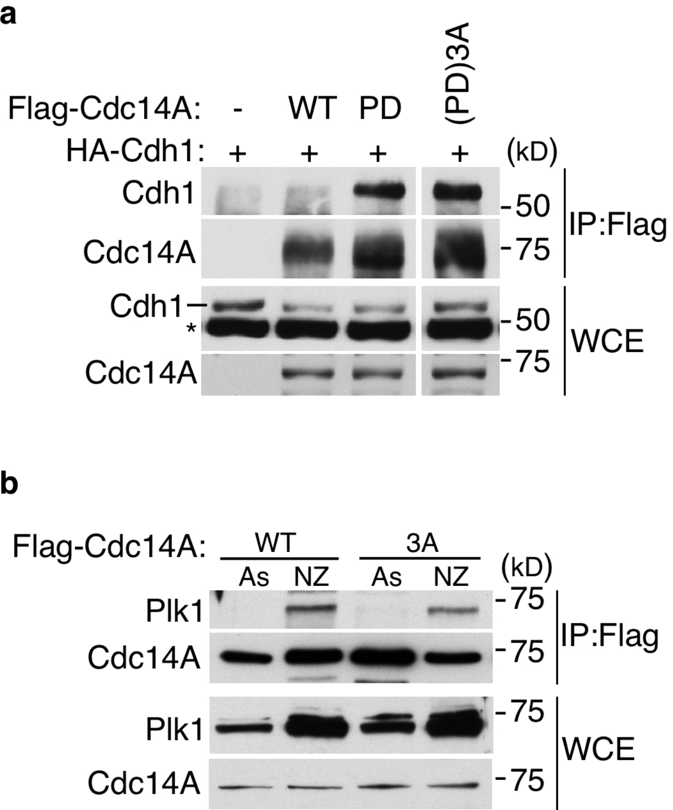

1a

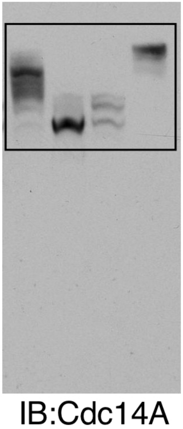
IB:Cdc14A

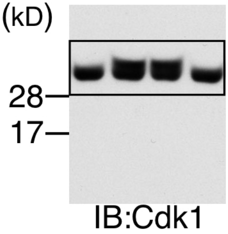
(kD)
28
17
IB:Cdk1

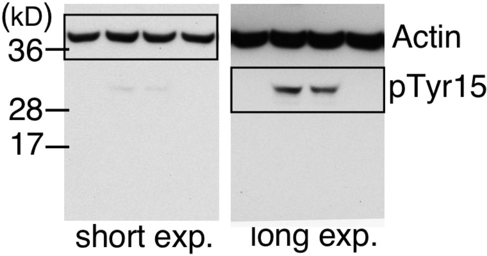
(kD)
36
28
17
Actin
pTyr15
short exp.
long exp.

1b

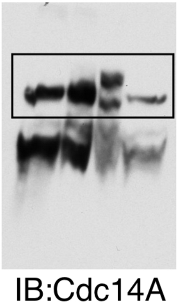
IB:Cdc14A

Supplementary Figure S10

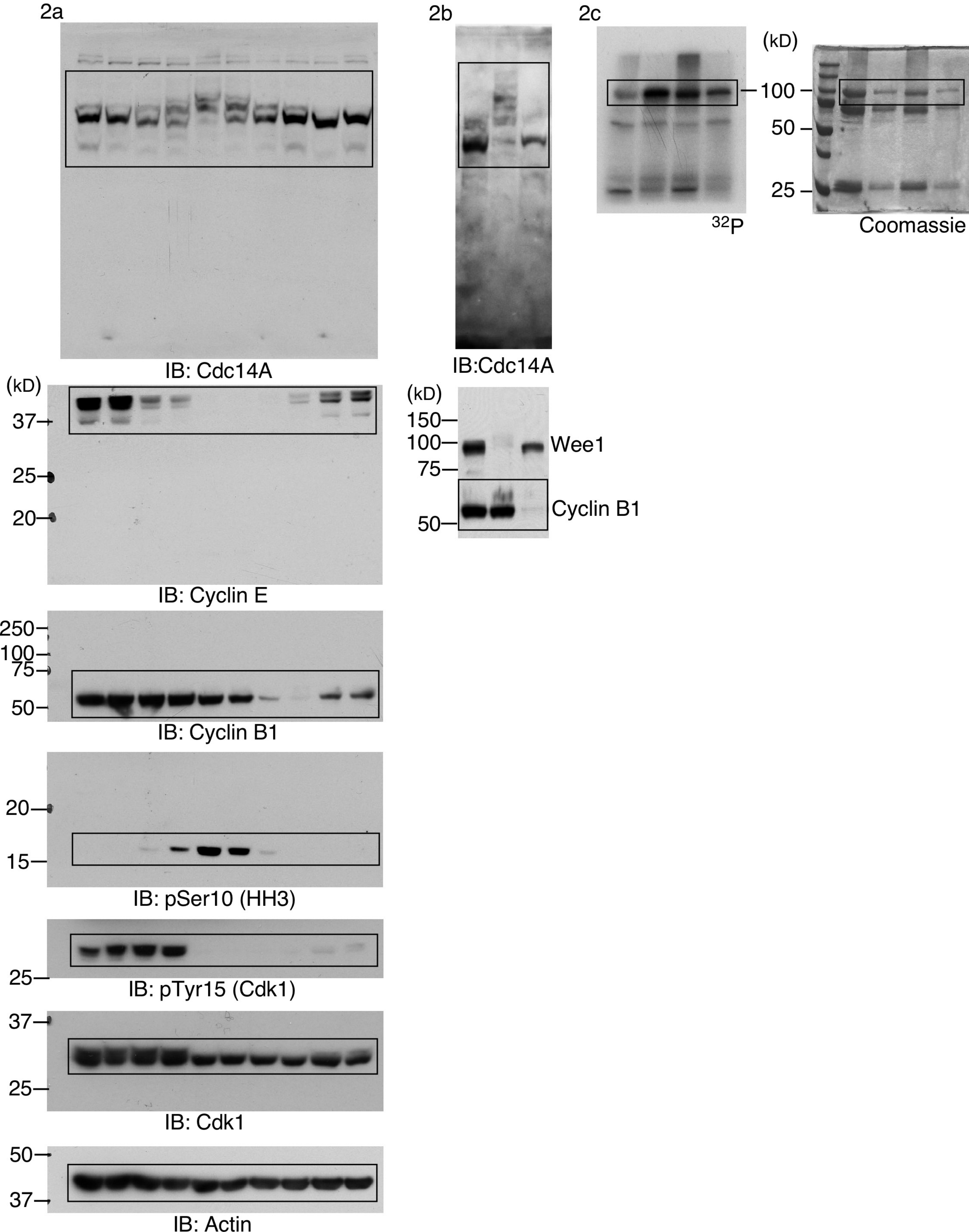

Supplementary Figure S11

3a

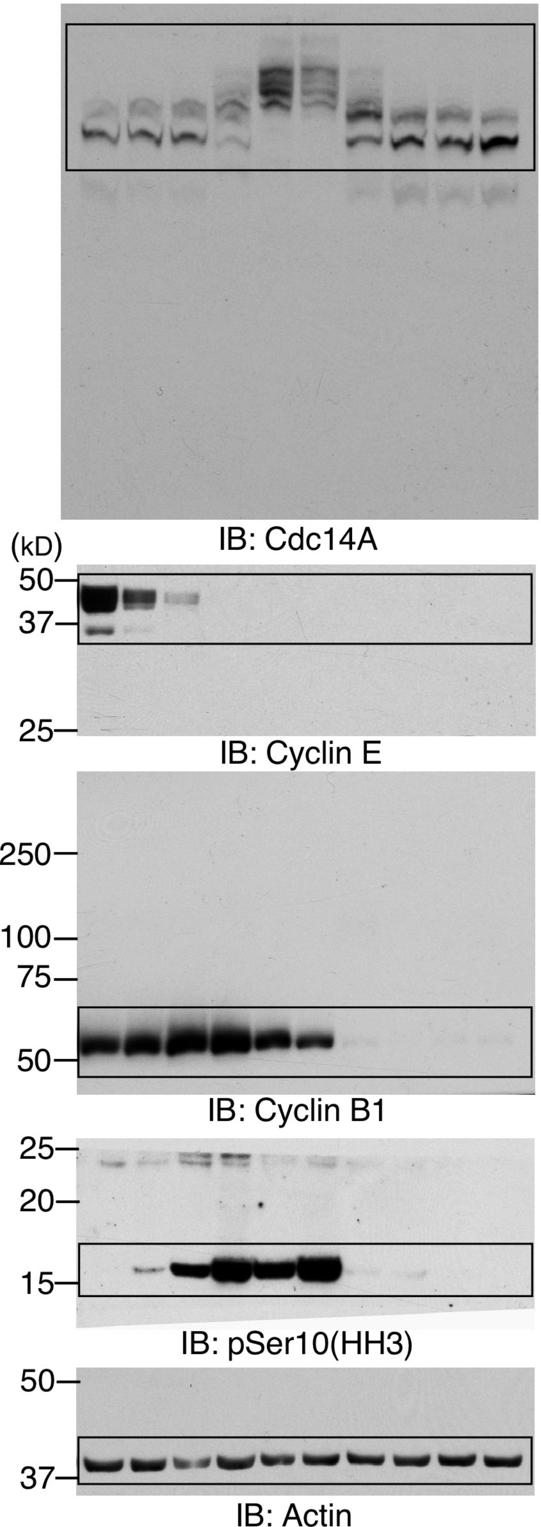

3b

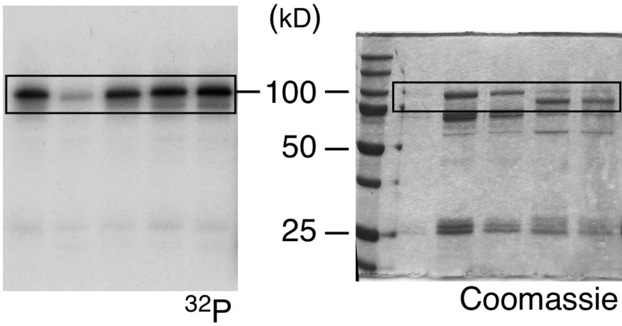

3c

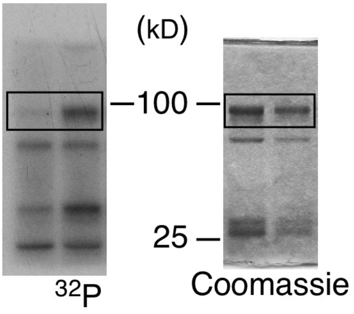

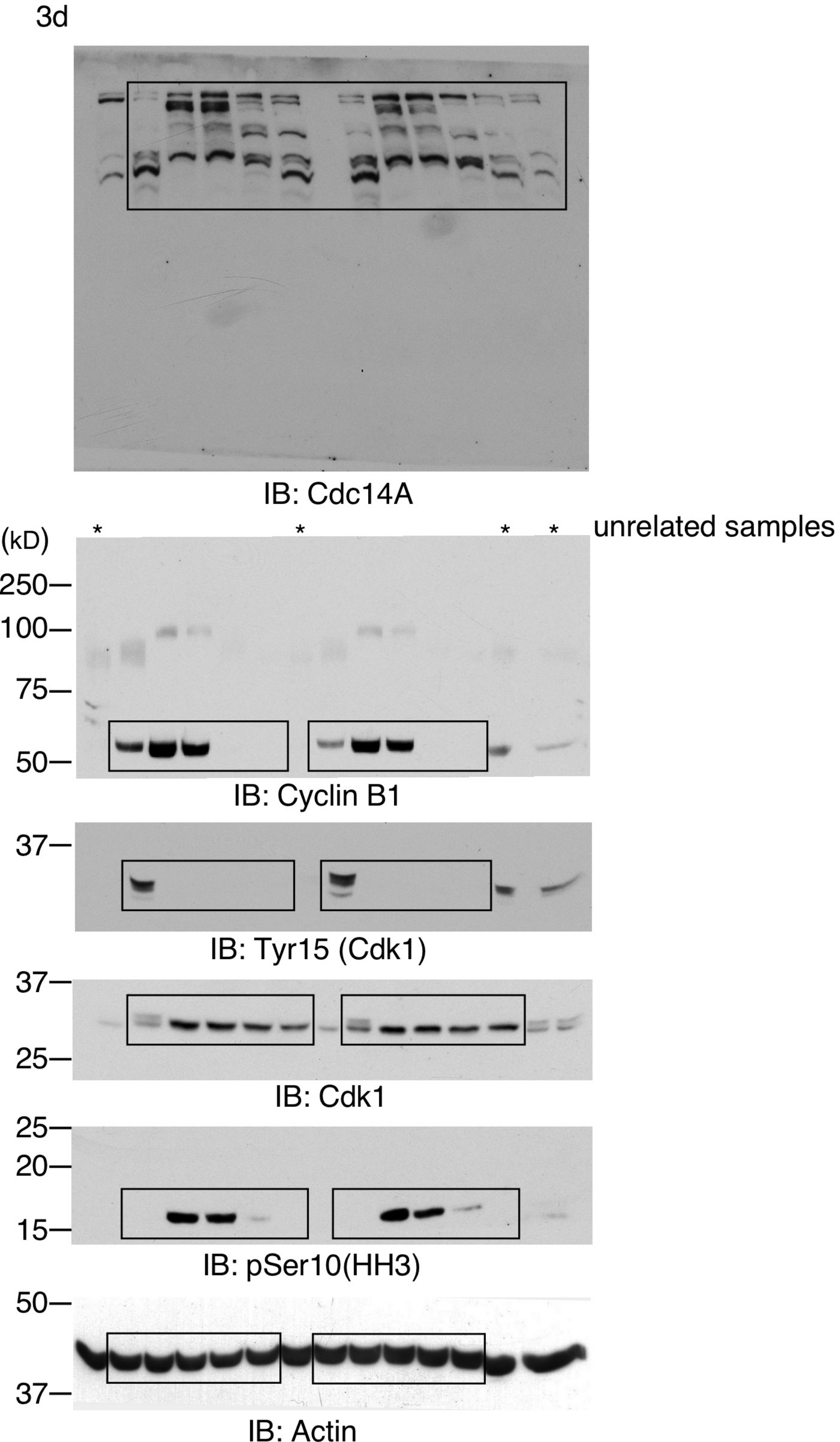
3d
IB: Cdc14A
unrelated samples
(kD)
250—
100—
75—
50—
IB: Cyclin B1
37—
IB: Tyr15 (Cdk1)
37—
25—
IB: Cdk1
25—
20—
15—
IB: pSer10(HH3)
50—
37—
IB: Actin

4b

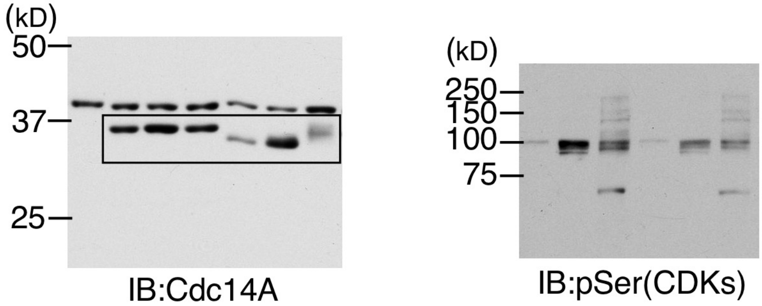
(kD)
50
37
25
IB:Cdc14A
(kD)
250
150
100
75
IB:pSer(CDKs)

4d

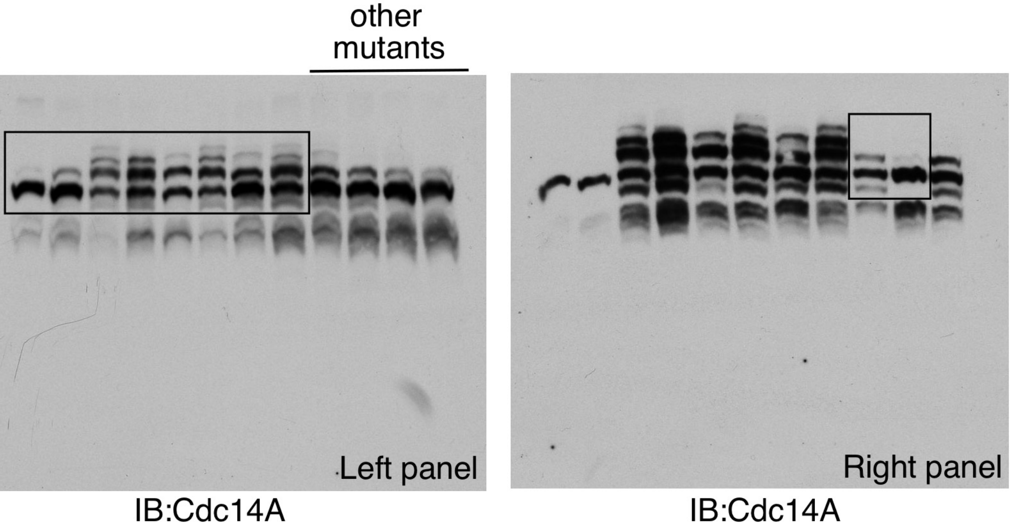
other mutants
Left panel
IB:Cdc14A
Right panel
IB:Cdc14A

4e

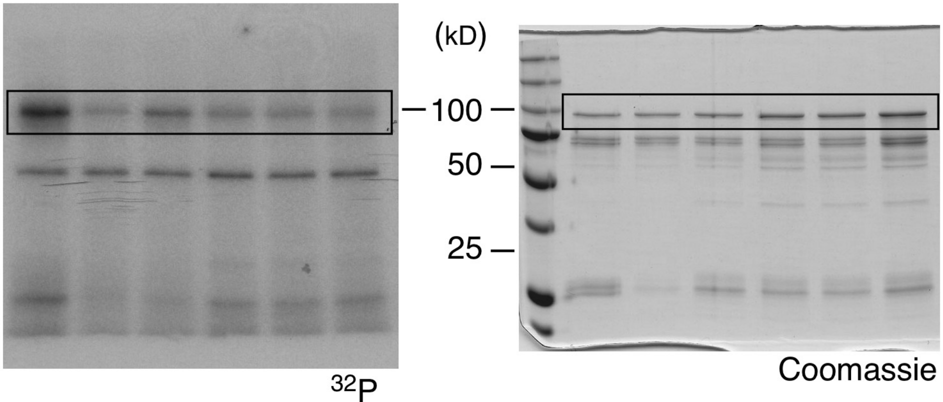
^{32}P
(kD)
100
50
25
Coomassie

5a

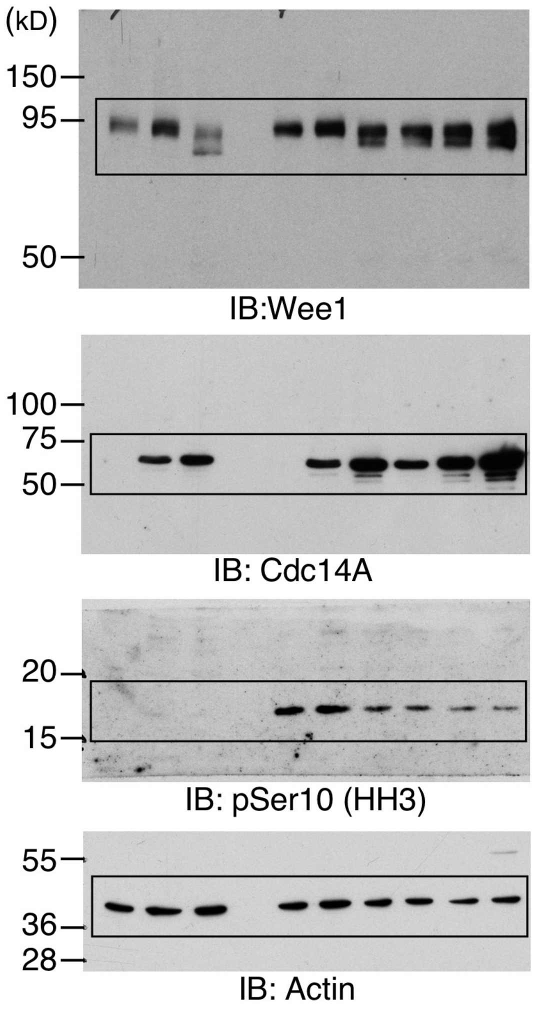
(kD)
150
95
50
IB:Wee1
100
75
50
IB: Cdc14A
20
15
IB: pSer10 (HH3)
55
36
28
IB: Actin

5b

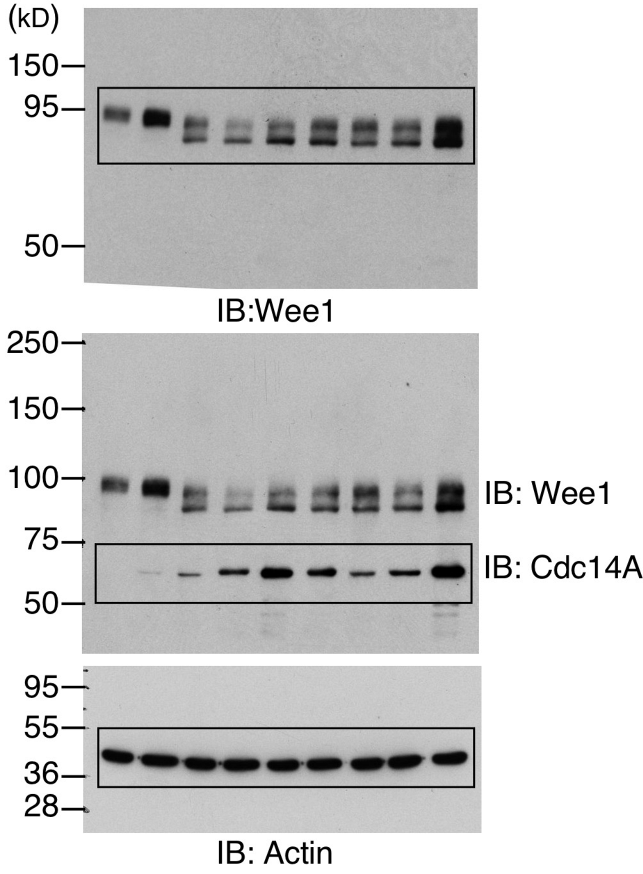
(kD)
150
95
50
IB:Wee1
250
150
100
75
50
IB: Wee1
IB: Cdc14A
95
55
36
28
IB: Actin

5c

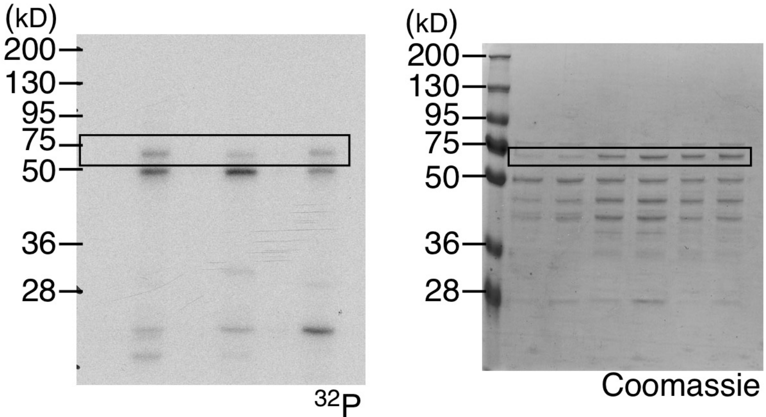
(kD)
200
130
95
75
50
36
28
32P
(kD)
200
130
95
75
50
36
28
Coomassie

6b

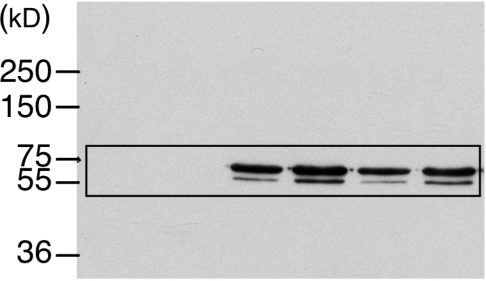

IB: Cdc14A

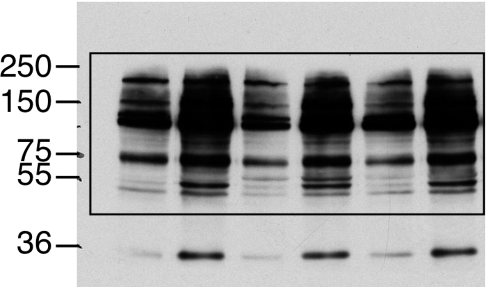

IB: pSer (CDKs)

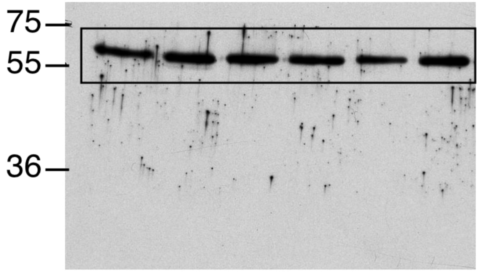

IB: Cyclin B1

Supplementary Figure S15

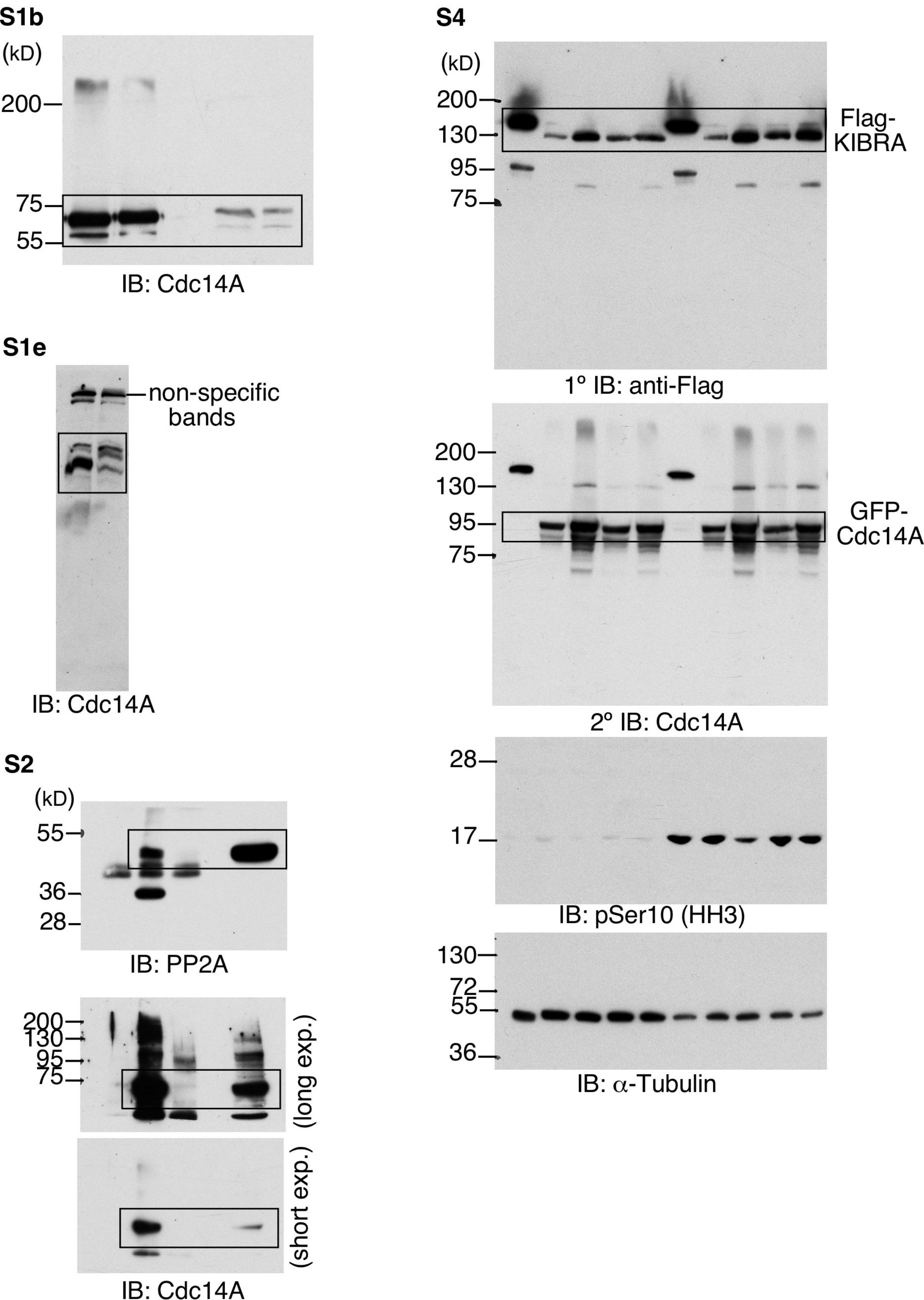

Supplementary Figure S16

S7

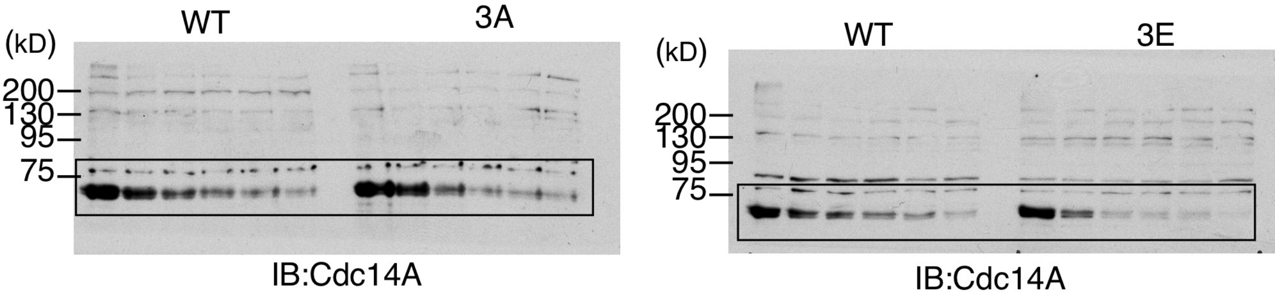

S8a

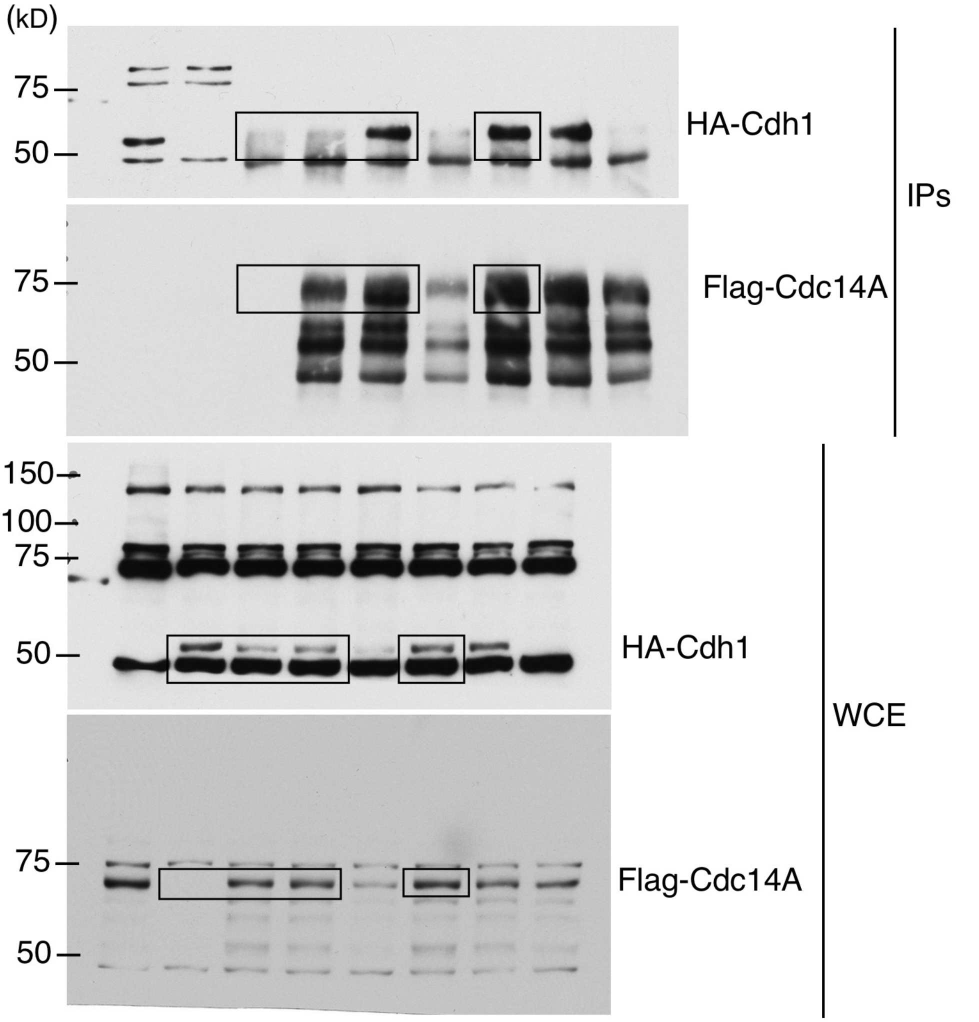

S8b

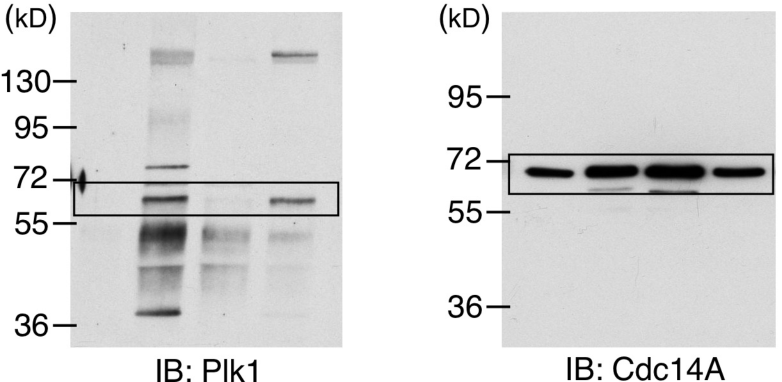

Supplement: Supplementary file 1 — Supplementary information [file 41598_2018_30253_MOESM1_ESM.pdf]
